# Chemical Composition and Anti-Candidiasis Mediated Wound Healing Property of *Cymbopogon nardus* Essential Oil on Chronic Diabetic Wounds

**DOI:** 10.3389/fphar.2016.00198

**Published:** 2016-06-30

**Authors:** Raghuram Kandimalla, Sanjeeb Kalita, Bhaswati Choudhury, Suvakanta Dash, Kasturi Kalita, Jibon Kotoky

**Affiliations:** ^1^Drug Discovery Lab, Institute of Advanced Study in Science and TechnologyGuwahati, India; ^2^Girijananda Chowdhury Institute of Pharmaceutical ScienceGuwahati, India; ^3^Pathology Department, Hayat HospitalGuwahati, India

**Keywords:** diabetic wound, *Candida albicans*, *Cymbopogon nardus*, cytokines, wound healing

## Abstract

Poor wound healing is one of the major complication of diabetic patients which arises due to different factors like hyperglycemia, oxidative stress, vascular insufficiency and microbial infections. Candidiasis of diabetic wounds is a difficult to treat condition and potentially can lead to organ amputation. There are a few number of medications available in market to treat this chronic condition; which demands for alternative treatment options. In traditional system of medicine like Ayurveda, essential oil extracted from leaves of *Cymbopogon nardus* L. (Poaceae) has been using for the treatment of microbial infections, inflammation and pain. In this regard, we have evaluated anti-*Candida* and anti-inflammatory activity mediated wound healing property of *C. nardus* essential oil (EO-CN) on candidiasis of diabetic wounds. EO-CN was obtained through hydro-distillation and subjected to Gas chromatography–mass spectroscopy (GC–MS) analysis for chemical profiling. Anti-*Candida* activity of EO-CN was tested against *Candida albicans*, *C. glabrata* and *C. tropicalis* by *in vitro* zone of inhibition and minimum inhibitory concentration (MIC) assays. Anti-candidiasis ability of EO-CN was evaluated on *C. albicans* infected diabetic wounds of mice through measuring candida load on the 7th, 14th, and 21st day of treatment. Further progression in wound healing was confirmed by measuring the inflammatory marker levels and histopathology of wounded tissues on last day of EO-CN treatment. A total of 95 compounds were identified through GC–MS analysis, with major compounds like citral, 2,6-octadienal-, 3,7-dimethyl-, geranyl acetate, citronellal, geraniol, and citronellol. *In vitro* test results demonstrated strong anti-*Candida* activity of EO-CN with a MIC value of 25 μg/ml against *C. albicans*, 50 μg/ml against *C. glabrata* and *C. tropicalis.* EO-CN treatment resulted in significant reduction of candida load on diabetic wounds. Acceleration in wound healing was indicated by declined levels of inflammatory cytokines at wounded area in EO-CN treated animals compared to non-treated group, which was further confirmed by histopathological examination. This study suggests that through significant anti-*Candida* and anti-inflammatory activity, EO-CN attenuates the growth of the fungus on diabetic wounds and simultaneously reduces the inflammation which leads to acceleration of the wound healing process.

## Introduction

Diabetes is a metabolic disorder associated with significantly increased blood sugar levels. Prolonged and untreated diabetes leads to chronic conditions like neuropathy, nephropathy, retinopathy, and cardiomyopathy. Wound development occurs in around 15% of diabetic patients and 70% of these wounds remain unhealed up to 20 weeks of drug treatment. Delay in wound healing in diabetic patients is due to factors like hyperglycemia, oxidative stress, vascular insufficiency, and chronic microbial infections (Gu et al., 2013). Besides bacterial infections, diabetic wounds are likely to be further complicated by an incidence of fungal infections such as candidiasis of interdigital spaces and toenails. *Candida* species is the most common yeast that infects diabetic wounds which lead to the delay in wound healing process (Emilija et al., 2005).

Further, *Candida* species are also responsible for infections of the blood stream, oral mucosa, and vaginal epithelium. *Candida albicans* is the most virulent pathogen among all *Candida* species which causes 40% candidemia related infections (Trofa et al., 2008). Limitations in market available drugs for the treatment of candidiasis and other dermatophytic wound infections of immunocompromised patients; demands alternative treatment options. Most of the antimicrobial, anti-inflammatory, and anticancer drugs available in the market are derived from the natural origin (Sarwar et al., 2015; Choudhury B. et al., 2016; Kandimalla et al., 2016a).

*Cymbopogon nardus* L. (Poaceae) grass is cultivated in large quantity in countries like India, Sri Lanka, and Malaysia for extracting the valuable essential oil. The oil has been used traditionally as an antimicrobial, antispasmodic, rubefacient, stimulant, insect repellent, carminative, and diaphoretic agent (Sinha et al., 2011). It is also widely used in the perfume industry and soap manufacturing process (Man et al., 2012). EO-CN was reported to have strong broad spectrum antifungal (Li et al., 2013) and anti-bacterial activity against systemic bacterial infections of aquatic animals (Wei and Wee, 2013). In this study, we for the first time demonstrated the novel therapeutic role of EO-CN on candidiasis of diabetic wounds. Further, the absence of a suitable animal model, to evaluate the effectiveness of potential therapeutic agents against infections of the diabetic wound has hindered to perform preclinical experimentation of such herbal remedies. Therefore, the present study was undertaken with two basic aims: (i) To develop an ideal animal model for evaluation of potential drug candidates against fungal infections of the diabetic wound. (ii) To evaluate the wound healing efficacy of EO-CN against candidiasis of diabetic wound.

## Materials and Methods

### Chemicals and Reagents

The gas chromatography–mass spectroscopy (GC–MS) solvents procured from Fischer scientific, USA. Microbial culture media obtained from Himedia laboratories, India. All the ELISA kits purchased from R&D systems, USA. All the other standard drugs and chemicals were of analytical grade and obtained from Sigma–Aldrich Co, St Louis, USA.

### Extraction of Essential Oil

Leaves of *C. nardus* L. were collected from the medicinal plant garden of Institute of Advanced Study in Science and Technology (IASST) (26.1471 and 91.7356), Kamrup district of Assam, India in the month of January 2014. The plant was identified by a taxonomist at North East India Ayurvedic Institute (NEIAI) and a plant voucher specimen (IASST/MAP14-55) was deposited in the herbarium library of life sciences division, IASST. The leaves (1000 g) were hydro-distillated in a Clevenger-type apparatus using 4–5 L of deionized water for 3 h. The light yellowish green colored, pleasant scented essential oil was collected and stored in amber colored vials at 4°C for further use.

### GC–MS Analysis of EO-CN

The gas chromatography–mass spectroscopy (GC–MS) analysis was conducted in full scan acquisition mode on GC–MS TQ8030, Shimadzu, Japan (triple quadruple) instrument. The carrier gas used was helium (99.99%) at a flow rate of 1.1 ml/min and ran for 60 min. The capillary column, EB-5MS of 30 m length, 0.25 μm thickness, and 0.25 mm diameter was used for the analysis. The mass transfer line temperature and the source were kept at 310°C, and 230°C, respectively. The chemical constituents of the essential oil were identified by comparing the spectra with the NIST11 database.

### *In vitro* Anti-Candida Activity of EO-CN

#### Microbial Strains and Fungal Spore Suspension Preparation

*Candida albicans* (MTCC 3958), *C. glabrata* (MTCC 3984), and *C. tropicalis* (MTCC 1000) were procured from Institute of Microbial Technology (IMTECH), Chandigarh, India. Fungal strains were inoculated on Sabouraud dextrose agar (SDA) and incubated for 48 h at 28 ± 2°C. For the preparation of inoculum, 3–4 days old culture was scraped with a sterile loop and macerated in sterile saline solution (0.85%). The final spore suspension was adjusted to 1 × 10^6^ CFU/mL with saline.

#### Agar Well Diffusion Method

The antifungal assay was carried out by agar well diffusion method (Bora, 2016; Choudhury et al., 2016a,b). EO-CN was reconstituted in dimethyl sulfoxide (DMSO) at different concentrations (20–100 μg/ml) to evaluate the anti-*Candida* activity. The Sabouraud dextrose agar (SDA) plates were inoculated with spore suspension (1 × 10^6^ CFU/mL) of *C. albicans, C. glabrata* and *C. tropicalis* separately. The test sample was placed in the 6 mm agar well, made by cork borer and then incubated at 27°C for 3–4 days. EO-CN free DMSO was used as control and broad-spectrum antifungal drug Clotrimazole was used as standard. The growth inhibition zone visible around the well was considered as anti-*Candida* activity. All the results were expressed in mean ± standard deviation (SD) of tests performed in triplicate.

#### Minimum Inhibitory Concentration (MIC)

Minimum inhibitory concentration was determined according to the method described earlier (Sharma et al., 2011; Kalita S. et al., 2016) by adding various concentrations of essential oil (6.25–100 μg) in Sabouraud dextrose broth medium. Further, 100 μl of *Candida* inoculum was added to each tube and incubated the tubes at 28°C for 7 days. The MIC was regarded as the lowest concentration of the oil that did not permit any visible growth after 7 days of incubation.

### Effect of EO-CN on *C. albicans* Infected Diabetic Wounds of Mice

#### Development of Mice Models to Mimic Fungal Infection on Diabetic Wounds

##### Animals

Swiss albino mice weighing between 22 and 25 g were obtained from Pasteur Institute, Shillong and maintained in the animal house at Institute of Advanced Study in Science and Technology (IASST), Guwahati, Assam. Mice were housed for a week for acclimatization before the initiation of the experiment in polypropylene cages and fed with rodent pellet diet (obtained from Provimi Animal Nutrition Pvt. Ltd., India) and water *ad libitum*. The laboratory conditions were maintained as per the standard guidelines (temperature 22 ± 2°C, relative humidity 60–70%, and 12–12 h light–dark cycle). All the experiments were carried out between 09:00 and 17:00 h. The experimental protocol was approved (IASST/IAEC/2014-15/747) by the Institutional Animal Ethics Committee (IAEC) of IASST, Guwahati before starting the experiments and performed in accordance with the guidelines of Committee for Control and Supervision of Experimentation on Animals (CPCSEA), Government of India on animal experimentation.

### Induction of Diabetic Wound

Diabetes was induced in mice by single intraperitoneal (i.p.) injection of streptozotocin (STZ) dissolved in 0.1 M citrate buffer (pH 4.5) at a dose of 60 mg/kg body weight. Three days post administration of STZ, diabetes was confirmed by measuring the fasting blood glucose levels through collecting blood from tail vein (Gupta et al., 2012). Mice having fasting blood glucose levels above 250 mg/dl were considered as diabetic and included in the study. After confirmation of diabetes, mice were anesthetized with ketamine hydrochloride and xylazine cocktail at 80 and 10 mg/kg, respectively by intramuscular (i.m.) injection. The hair on the dorsal leg region was removed and sterilized with povidone iodine solution and full thickness excisional wound of 4 mm was created by using sterile biopsy punch. No dressing material was used to close the wounds throughout the study (Abu-Al-Basal, 2010).

### *Candida* Infection on Diabetic Wounds

Diabetic mice were anesthetized under light ether anesthesia and the wounds were infected with *C. albicans* inoculums. The fungal inoculums were prepared with 10^7^ *C. albicans* cell suspension of 3–4 day old culture slants by macerating with 0.25 g of sterilized white sand powder and 2.75 g of vaseline jelly. The fungal inoculum was applied on the diabetic wounds and the whole area was covered with cloth bandage and tied with nonirritant leucoplast. After 24 h, the bandage was removed and the wounded area was cleaned with sterile water. Further, animals were caged individually for 5 days with adequate supply of food and water.

### Experimental Design

After 5 days of observation period, animals were randomly divided into 3 groups (*n* = 6) for drug treatment:

*Group-I:* Mice with diabetic wound and treated with saline.
*Group-II:* Mice with diabetic wound infected with *C. albicans* and treated with saline.
*Group-III:* Mice with diabetic wound infected with *C. albicans* and treated with EO-CN topically (25 μg/day) dispersed in 100 μl of olive oil for 21 days daily.
*Group-IV*: Mice with diabetic wound infected with *C. albicans* and treated with Clotrimazole topically (1 μg/day) dispersed in 100 μl of olive oil for 21 days daily.

### Fungal Culture Recovery from Diabetic Wound

Effects of the EO-CN was evaluated by culturing skin scrapings from the infected areas of the mice in SDA plates on 7th, 14th, and 21st day of treatment period (Kalita et al., 2015). Percent culture recovery was calculated using the formula:

$$\%Culture\,\;re\,\mathrm{cov}ery=\frac{Total\,\;number\,\;of\,\;sites\,\;positive\,\;for\,\;culture\,\;in\,each\,\;set}{Total\,\;number\,\;of\,\;sites\,\;in\,\;each\,\;set}\times 100$$

### CFU Count Determination

On 7th, 14th, and 21st day of treatment period, diabetic wounds from different treatment groups were excised, pulverized and homogenized in sterile phosphate buffer saline. Tissue homogenates were diluted 100-fold and plated on SDA in order to tally the CFU count of *C. albicans* grown after 6 days of incubation at 28 ± 2°C. All results were normalized based on excised tissue weight.

### Tissue Harvesting and Measurement of TNF-α and IL-1β

After 21 days of treatment period, animals from all the groups were sacrificed by overdose of ether anesthesia and wounded tissues were collected in liquid nitrogen for further use. The frozen tissues (100 mg) were homogenized in 1 ml lysis buffer [1% Triton X, 10 mM phenylmethylsulfonyl fluoride (PMSF), 1 mg/ml aprotinin and 1 mg/ml leupeptin in phosphate buffer saline (PBS) pH 7.4] and the resulted homogenates were centrifuged at 12,000 rpm for 10 min at 4°C. Supernatant was collected separately and stored at -80°C till further use for ELISA testing (Kant et al., 2013). The levels of inflammatory cytokines TNF-α and IL-1β were measured by using ELISA kits from R&D systems, USA according to the instructions given by the manufacturers. Each sample was performed in duplicate and the results were expressed in Pg/ml.

### Histopathology of Wound

At the end of 21st day of treatment period, tissues from the wounded area were collected in 10% buffered formaldehyde and preserved for at least 24 h. Further, the tissues were dehydrated gradually with ethanol (70–100%), cleared in xylene and embedded in paraffin. Sections of 4–5 μm were prepared and stained with hematoxylin–eosin. The pathological changes were examined under light microscope (10×) to determine the difference in wound healing between the treatment groups (Bhardwaj et al., 2016).

### Statistical Analysis

All results are expressed as the mean ± standard deviation. For *Candida*, load measurement statistical analysis was performed using GraphPad Prism version 6.01, using one-way ANOVA followed by Bonferroni posttest for comparison between the groups. A *P* value < 0.05 was considered statistically significant.

## Results and Discussion

### Chemical Components of EO-CN

Hydro-distillation of *C. nardus* yields 3% of essential oil. A total of 95 compounds were identified from GC–MS analysis of EO-CN with principle compounds *viz*, citral, 2,6-octadienal-, 3,7-dimethyl-, geranyl acetate, citronellal, geraniol, and citronellol. Chemical composition along with its percentages of the EO-CN was shown in **Table 1**. In the present study, we have found EO-CN is mainly composed of terpenoids, monoterpenes, and monoterpenols. The chemical composition of EO-CN explored in this study matches with the earlier reported literature (Wijesekera, 1973; Nakahara et al., 2003; Ganjewala, 2009; Aguiar et al., 2014; Kpoviessi et al., 2014).

**Table 1:** GC–MS analysis of EO-CN showing chemical compounds with percentages.

S. No.	Chemical compound	Percentage (%)
1	Citral	38.75
2	2,6-Octadienal, 3,7-dimethyl-	31.02
3	Citronellal	6.06
4	Geranyl acetate	4.28
5	Geraniol	2.75
6	Citronellol	1.89
7	Trans-2-[2′-(2′′-methyl-1′′-propen	1.14
8	Caryophyllene oxide	1.12
9	Neric acid	1.00
10	Carane, 4,5-epoxy-, trans	0.88
11	1,6-Octadien-3-ol, 3,7-dimethyl	0.77
12	6-Octen-1-ol, 3,7-dimethyl-	0.71
13	1-Acetyl-2-(2’-oxo-propyl)-	0.63
14	Beta-copaene	0.62
15	7-Oxabicyclo[4.1.0]heptane	0.56
16	5-Hepten-2-one, 6-methyl-	0.54
17	4-Nonanone	0.47
18	Camphene	0.43
19	D-Limonene	0.40
20	Cubenol	0.30
21	Caryophyllene	0.27
22	Benzene, 1,3-bis(1,1-dimethyl)	0.21
23	Isobornyl acetate	0.20
24	Carane, 4,5-epoxy-	0.19
25	Alpha-cadinol	0.18
26	Endo-Borneol	0.17
27	6-Octenoic acid, 3,7-dimethyl-	0.17
28	5-Heptenal, 2,6-dimethyl-	0.16
29	6-Octen-1-ol, 7-methyl-3-	0.16
30	Dodecane, 2,6,11-trimethyl-	0.16
31	Cyclohexanemethanol	0.15
32	2-Buten-1-one, 1-	0.15
33	Decanal	0.12
34	12-Oxabicyclo[9.1.0]dodeca-3	0.11
35	7-Acetyl-2-hydroxy-2-methyl-	0.11
36	Heptadecane	0.10
37	2,10-Dodecadien-1-ol	0.10
38	Minor compounds	2.97

### *In vitro* Anti-*Candida* Activity of EO-CN

The EO-CN inhibits the growth of all the *Candida* species tested in a dose-dependent manner. EO-CN exhibited pronounced zone of inhibition against *C. albicans* with 62 mm zone at highest concentration (100 μg/ml) and 19 mm zone at lowest concentration (20 μg/ml) (**Table 2**). EO-CN inhibits the growth of *C. albicans* at lowest concentration with MIC value of 25 μg w/v and *C. glabrata*, *C. tropicalis* at 50 μg w/v. Among all the candida species, *C. albicans* is the most virulent fungal pathogen of chronic wound infection. The excellent fungicidal property shown by EO-CN against *C. albicans* establish this essential oil as a promising therapeutic option to treat candidiasis of chronic wounds. The oxygenated terpenes are reported as potent antifungal agents (Policegoudra et al., 2012) and presence of monoterpenes, terpenoids and monoterpenols in EO-CN might be responsible for the anti-*Candida* activity. This result clearly indicates the antagonistic effect of EO-CN against *Candida* species.

**Table 2:** Zone of inhibition and minimum inhibitory concentration (MIC) of EO-CN against *Candida* species.

S. No.	Fungal strain	Zone of inhibition (mm)						MIC (μg/ml) EO-CN	MIC (μg/ml) Clotrimazole
		20 μg/ml	40 μg/ml	60 μg/ml	80 μg/ml	100 μg/ml	Clotrimazole (20 μg/ml)		
1	*Candidaalbicans* (MTCC 3958)	19 ± 1.6	27 ± 1.4	39 ± 1.9	47 ± 1.4	62 ± 2.2	46 ± 1.6	25	1
2	*C. glabrata* (MTCC 3984)	17 ± 1.2	25 ± 1.1	36 ± 1.3	44 ± 1.3	56 ± 1.8	41 ± 1.3	50	1
3	*C. tropicalis* (MTCC 1000)	16 ± 1.4	24 ± 1.2	36 ± 1.6	43 ± 1.5	55 ± 2.0	44 ± 1.1	50	0.5

*All results are expressed in mean ± SD*.

### *C. albicans* Infection on Diabetic Wounds of Mice

Diabetes induction by STZ is a widely used method, which shows classical symptoms like hyperglycemia, polyphagia, polydipsia, and reduced body mass (Lima et al., 2012; Kalita H. et al., 2016). Diabetic wounds are more prone to microbial infections than normal wounds due to the high levels of blood glucose in the wound fluids that allows microbes to grow fast (Hirsch et al., 2008). To mimic this condition, in the present study we infected the diabetic wounds of mice with *C. albicans*. We observed that fungal infected wounds were not healed up to 21 days whereas non-infected diabetic wounds were healed by this period. Histopathology of wound showed wide area of necrosis with no signs of wound healing (Group-II) compared to normal diabetic wounds (Group-I). This animal model can be used to evaluate the wound healing property of phytochemical/drugs against fungal pathogens infected diabetic wounds.

### Effect of EO-CN on *C. albicans* Infected Diabetic Eounds

#### % Culture Recovery

**FIGURE 1:**
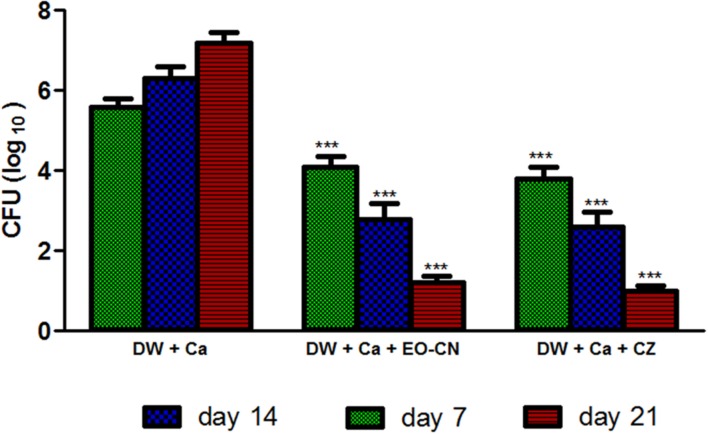
**CFU load of *C. albicans* on diabetic wounds treated with EO-CN and CZ (Group-III and IV) at 7th, 14th, and 21th day.** DW: Diabetic wounds; Ca: *Candida albicans*; EO-CN: Essential oil from *Cymbopogon nardus*; CZ: Clotrimazole. ^∗∗∗^*P* ≤ 0.001 in comparison of treatment groups with untreated group.

CFU data enumeration reveals that EO-CN treated group showed significantly low *Candida* load on wounded area than non-treated group, which is comparable with standard drug clotrimazole (**Figure 1**). EO-CN treatment effectively eradicated the *C. albicans* colonization on diabetic wounds on 7th, 14th, and 21st day of post-treatment (**Table 3**), which led to the acceleration in wound closure in comparison to the non-treated group. The qualitative clinical analysis reveals the efficacy of EO-CN treatment on *C. albicans* infected diabetic wound through decreased peripheral wound erythema, necrotic crusting and tissue induration with edema in comparison to non-treated group. *C. albicans* invades wounded tissue of diabetic patients by puncturing the skin with its hyphae. In diabetic condition, high levels of nutrients like sugar provide a favorable environment to the fungi to produce the spores and double the fungal population in 1 h. This overgrowth worsens the diabetic wounds and renders it difficult to cure by market available anti-fungal agents. Pronounced cutaneous penetration ability of essential oil can effectively eradicate the fungal structures through inhibiting fungal spore formation and hyphal elongation which in turn obstructs the disease progression. The major components of EO-CN were citral, citronellal, geranyl acetate, geraniol, and citronellol was reported to have broad spectrum antimicrobial activity (Zore et al., 2011) which is responsible for protecting the wounded area from *C. albicans* infection.

**Table 3:** Effect of EO-CN and clotrimazole treatment on % culture recovery of *C. albicans* from diabetic wounds on different time intervals (7th, 14th, and 21st day).

S. No.	Treatment days	% Culture recovery		
		Control	EO-CN	Clotrimazole
1	7th	100	82.34 ± 3.67	77.82 ± 3.24
2	14th	100	38.28 ± 2.12	34.72 ± 2.48
3	21st	100	3.64 ± 0.36	2.14 ± 0.19

### Effect of EO-CN on Inflammatory Cytokines Levels

**FIGURE 2:**
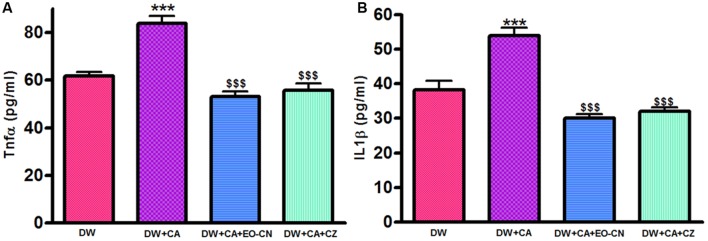
**Effect of EO-CN and CZ treatment on TNF-α (A) and IL-1β (B) levels.** All the results were expressed in mean ± SD. ^∗∗∗^*P* ≤ 0.001 comparison of *C. albicans* infected diabetic wounds with uninfected diabetic wounds. DW: Diabetic wounds; Ca: *C. albicans*; EO-CN: Essential oil from *C. nardus*; CZ: Clotrimazole. ^$$$^*P* ≤ 0.001 comparison of EO-CN and CZ treated *C. albicans* infected diabetic wounds with untreated *C. albicans* infected diabetic wounds.

*Cymbopogon albicans* infection cause significant (*p* ≤ 0.001) rise in inflammatory cytokines like TNF-α and IL-1β levels in wounded area compared to non-infected diabetic wounds. Twenty-one days of EO-CN and standard drug clotrimazole treatment on *C. albicans* infected diabetic wounds showed reduced levels of these inflammatory markers compared to non-treated animals (**Figures 2A,B**). Major chemical constitutes of EO-CN like citral, citronellal, geranyl acetate, geraniol, and citronellol possesses remarkable anti-inflammatory ability which acts through the stabilization of macrophages (Bachiega and Sforcin, 2011; Jayachandran et al., 2015; Kant et al., 2015). Decreased levels of inflammatory cytokines are the indication of reduction of infection as well as progression of wound healing and repair (Kandimalla et al., 2016b). EO-CN possess both anti-fungal and anti-inflammatory property which not only eradicate the fungal infection but also reduce the inflammation and its associated complications.

### Histopathology

**FIGURE 3:**
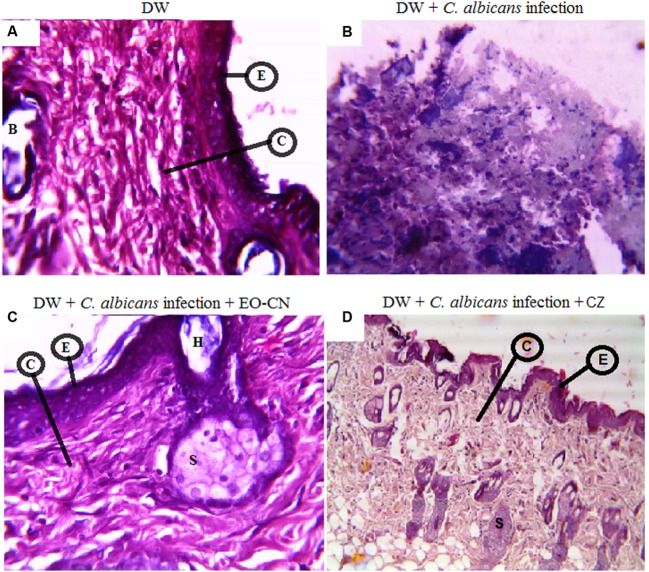
**Histopathology analysis of diabetic wounds of mice post 21 days of treatment. (A)** Normal diabetic wounds with no infection. **(B)** *C. albicans* infected diabetic wounds. **(C)** *C. albicans* infected diabetic wounds treated with EO-CN. **(D)** *C. albicans* infected diabetic wounds treated with CZ. Abbreviations: E: Epidermis; C: Collagenase tissue; B: Blood vessel; H: Hair follicles; S: Sweat glands; DW: Diabetic wounds; EO-CN: Essential oil from *C. nardus*; CZ: Clotrimazole.

The histopathological examination of the skin collected from diabetic wounds of mice in group-I on the 21st day showed fragments of dermal tissue lined by atrophic epidermis with flattened rete ridges (**Figure 3A**). On infection with *C. albicans*, the diabetic wounds were not healed until 21 days which showed fragments of degenerated tissue with foci of adipose tissue and widespread necrosis (**Figure 3B**). Treatment with EO-CN on *C. albicans* infected diabetic wounds completely healed the wounds which were confirmed by observation of fibro collagenase subepidermal tissue with normal skin adnexal structures like hair follicles and sweat glands with mild cell infiltrate (**Figure 3C**) which is comparable with standard drug clotrimazole (**Figure 3D**). This result confirms the wound healing ability of EO-CN through inhibiting the fungal growth on diabetic wounds.

## Conclusion

Diabetic wounds are chronic in nature and susceptible to microbial infections. In this study, we developed an animal model which mimics fungal infected diabetic wounds. This model can be used for therapeutic evaluation of potential drug candidates to overcome the pathophysiological complexities arising in treatment of fungal pathogen infected diabetic wounds. This study suggests that EO-CN possesses anti-fungal and anti-inflammatory chemical constituents which can be used topically to prevent candidiasis on chronic diabetic wounds. Feasibility of application of EO-CN as a dermal therapeutic agent can potentially lead to its commercialization with further pharmaceutical developments.

## Author Contributions

RK designed and performed the whole study, he developed the new animal model and wrote the manuscript. SK performed the antimicrobial tests and helped Mr. RK in all the tests conducted and manuscript preparation. BC helped in anti-fungal studies. KK performed and analysed the hisopathology samples. SD helped in study design and results analysis. JK supervised the study, complied the results and corrected the manuscript.

## Disclaimer

A portion of the work reported in this article was used to file an Indian patent application number (287/KOL/2015) dated 18th March, 2015. This filed application was published in official journal of Indian patent office on 17th April, 2015.

## Conflict of Interest Statement

The authors declare that the research was conducted in the absence of any commercial or financial relationships that could be construed as a potential conflict of interest.
